# Supplementary material for: A Novel Risk Score Model of Lactate Metabolism for Predicting over Survival and Immune Signature in Lung Adenocarcinoma
Source: Cancers (Basel). 2022 Jul 30;14(15):3727. doi: 10.3390/cancers14153727 (PMC9367335; doi:10.3390/cancers14153727)
Supplement: Supplementary file 1 [file cancers-14-03727-s001.zip › cancers-1731224-supplmentary.pdf]

Table S1. 24 lactate metabolism-associated genes.

**Genes**
LIAS
BCKDHB
DDC
ABAT
BCKDHA
SLC10A2
DBT
PUS1
LDHD
SLC17A5
DLD
YARS2
PC
PDHA1
GAA
LRPPRC
DLAT
PDHB
MT-ATP6
MT-ND1
MT-ND5
MT-ND4
MT-ND6
MT-CO1
